# Characterization of Oseltamivir-Resistant 2009 H1N1 Pandemic Influenza A Viruses

**DOI:** 10.1371/journal.ppat.1001079

**Published:** 2010-08-26

**Authors:** Maki Kiso, Kyoko Shinya, Masayuki Shimojima, Ryo Takano, Kei Takahashi, Hiroaki Katsura, Satoshi Kakugawa, Mai thi Quynh Le, Makoto Yamashita, Yousuke Furuta, Makoto Ozawa, Yoshihiro Kawaoka

**Affiliations:** 1 Division of Virology, Department of Microbiology and Immunology, Institute of Medical Science, University of Tokyo, Shirokanedai, Minato-ku, Tokyo, Japan; 2 Division of Zoonosis, Department of Microbiology and Infectious Diseases, Graduate School of Medicine, Kobe University, Hyogo, Japan; 3 National Institute of Hygiene and Epidemiology, Hanoi, Vietnam; 4 Daiichi Sankyo Co Ltd, Shinagawa, Tokyo, Japan; 5 Toyama Chemical Co., Ltd., Toyama, Japan; 6 International Research Center for Infectious Diseases, Institute of Medical Science, University of Tokyo, Shirokanedai, Minato-ku, Tokyo, Japan; 7 Department of Pathobiological Sciences, University of Wisconsin, Madison, Wisconsin, United States of America; 8 ERATO Infection-Induced Host Responses Project, Japan Science and Technology Agency, Saitama, Japan; Harvard Medical School, United States of America

## Abstract

Influenza viruses resistant to antiviral drugs emerge frequently. Not surprisingly, the widespread treatment in many countries of patients infected with 2009 pandemic influenza A (H1N1) viruses with the neuraminidase (NA) inhibitors oseltamivir and zanamivir has led to the emergence of pandemic strains resistant to these drugs. Sporadic cases of pandemic influenza have been associated with mutant viruses possessing a histidine-to-tyrosine substitution at position 274 (H274Y) in the NA, a mutation known to be responsible for oseltamivir resistance. Here, we characterized *in vitro* and *in vivo* properties of two pairs of oseltaimivir-sensitive and -resistant (possessing the NA H274Y substitution) 2009 H1N1 pandemic viruses isolated in different parts of the world. An *in vitro* NA inhibition assay confirmed that the NA H274Y substitution confers oseltamivir resistance to 2009 H1N1 pandemic viruses. In mouse lungs, we found no significant difference in replication between oseltamivir-sensitive and -resistant viruses. In the lungs of mice treated with oseltamivir or even zanamivir, 2009 H1N1 pandemic viruses with the NA H274Y substitution replicated efficiently. Pathological analysis revealed that the pathogenicities of the oseltamivir-resistant viruses were comparable to those of their oseltamivir-sensitive counterparts in ferrets. Further, the oseltamivir-resistant viruses transmitted between ferrets as efficiently as their oseltamivir-sensitive counterparts. Collectively, these data indicate that oseltamivir-resistant 2009 H1N1 pandemic viruses with the NA H274Y substitution were comparable to their oseltamivir-sensitive counterparts in their pathogenicity and transmissibility in animal models. Our findings highlight the possibility that NA H274Y-possessing oseltamivir-resistant 2009 H1N1 pandemic viruses could supersede oseltamivir-sensitive viruses, as occurred with seasonal H1N1 viruses.

## Author Summary

Although most of the currently circulating 2009 pandemic influenza A (H1N1) viruses are susceptible to neuraminidase (NA) inhibitors (oseltamivir and zanamivir), oseltamivir-resistant mutants have sporadically appeared. Yet, the pathogenicity and transmissibility of these oseltamivir-resistant 2009 H1N1 pandemic viruses remain unknown. Here, we compared the pathogenicity and transmissibility of two pairs of oseltamivir-sensitive and -resistant viruses in mouse and ferret models. We found that the oseltamivir-resistant viruses efficiently replicated in the lungs of mice treated with oseltamivir or even zanamivir. Further, we demonstrated that these oseltamivir-resistant viruses caused lung lesions in ferrets and efficiently transmitted between ferrets, as did their oseltamivir-sensitive counterparts. Overall, our results suggest the possibility that oseltamivir-resistant viruses could spread among humans without loss of pathogenicity.

## Introduction

Since its emergence in early spring of 2009, 2009 pandemic influenza A (H1N1) viruses have been circulating worldwide [1]. Although most infected individuals have exhibited an uncomplicated, mild respiratory disorder, the pathogenicity of this virus is clearly higher than that of seasonal influenza viruses in animal models [2]–[4] and humans, including those who do not have underlying illness [5].

Although two classes of anti-influenza drugs – M2 ion channel blockers (amino-adamantines; amantadine and rimantadine) and neuraminidase (NA) inhibitors (oseltamivir and zanamivir) – are licensed, the 2009 H1N1 pandemic viruses, including the earliest isolate, are already amino-adamantine-resistant [1]. By contrast, most of the currently circulating pandemic viruses are susceptible to NA inhibitors [2], [6], and therefore, pandemic influenza patients are treated with NA inhibitors in many countries. This widespread administration, however, raises concerns over the emergence and global spread of NA inhibitor-resistant 2009 H1N1 pandemic viruses.

Studies with seasonal H1N1, H3N2, and highly pathogenic avian H5N1 viruses revealed that single amino acid substitutions at several positions in or around the NA active site confer resistance to viruses against NA inhibitors [7]–[11]. Among these NA substitutions, a histidine-to-tyrosine substitution at position 274 (N2 numbering, H274Y) is one of the best characterized oseltamivir-resistant markers. Recently, the NA H274Y substitution has been detected in sporadic cases of oseltamivir-treated and -untreated patients infected with 2009 H1N1 pandemic viruses [12]–[14]: however, the pathogenicity and transmissibility of viruses possessing this NA substitution remain unknown.

To better assess the risk of the 2009 H1N1 pandemic viruses that possess the NA H274Y substitution, we studied the *in vitro* and *in vivo* properties of two NA H274Y-possessing isolates that emerged independently: A/Osaka/180/2009 (H1N1; O180r) and A/Vietnam/HN32060/2009 (H1N1; VN32060r).

## Results/Discussion

### *In vitro* sensitivity of viruses to NA inhibitors

To test the *in vitro* sensitivity to various NA inhibitors of 2009 H1N1 pandemic viruses possessing the NA H274Y substitution, we measured the NA activity of O180r and VN32060r exposed to oseltamivir carboxylate (the active metabolite of oseltamivir), zanamivir, or R-125489 (the active metabolite of the experimental NA inhibitor CS-8958) and determined IC_50_ values. These IC_50_ values were compared with those of A/Osaka/164/2009 (O164s) and A/Vietnam/HCM9727/2009 (VN9727s), both of which do not possess the NA substitution (Supplementary Table S1) and thus served as control strains (Table 1). O164s and O180r were isolated on similar dates, circulating in the same community and were genetically similar. Likewise, VN9727s and VN32060r were similar with respect to their isolation history (temporally and geographically) and their genetic properties. The IC_50_ values of O180r and VN32060r were about 500-fold higher than those of O164s and VN9727s for oseltamivir, while the sensitivity to zanamivir and R-125489 was comparable for all viruses tested.

**Table 1:** *In vitro* virus susceptibility to NA inhibitors.

NA inhibitors	IC_50_ c			
	O164s	O180r	VN9727s	VN32060r
Oseltamivir carboxylate^a^	1.6^c^	856	1.63	811
Zanamivir	0.43	0.75	0.3	0.56
R-125489^b^	0.44	0.89	0.26	0.5

a Oseltamivir carboxylate is the active form of oseltamivir.

b R-125489 is the active form of CS-8958.

c IC_50_ value: 50% inhibitory concentration (nM) of duplicate reactions.

To further assess the *in vitro* sensitivity of oseltamivir-resistant 2009 H1N1 pandemic viruses to the NA inhibitors, we investigated the growth kinetics of the two pairs of oseltaimivir-sensitive and -resistant viruses in cultured cells in the presence of oseltamivir carboxylate, zanamivir, or R-125489 (Supplementary Fig. S1). While the growth of O164s and VN9727s was severely impaired by oseltamivir carboxylate, O180r and VN32060r replicated efficiently even in the presence of oseltamivir carboxylate. By contrast, both zanamivir and R-125489 inhibited the replication of all of the viruses tested. These results indicate that the NA H274Y substitution reduces the sensitivity of 2009 H1N1 pandemic viruses to oseltamivir, as has been shown with seasonal H1N1, H3N2, and highly pathogenic avian H5N1 viruses [7]–[11], [15], since the other amino acid variations among the viruses tested (Supplementary Table S1) did not account for their reduced oseltamivir sensitivity: the lower growth of O180r compared to that of O164s in the absence of antivirals (Supplementary Fig. S1) might be due to these additional amino acid differences.

### Replication and antiviral sensitivity of viruses in mice

**Figure 1:**
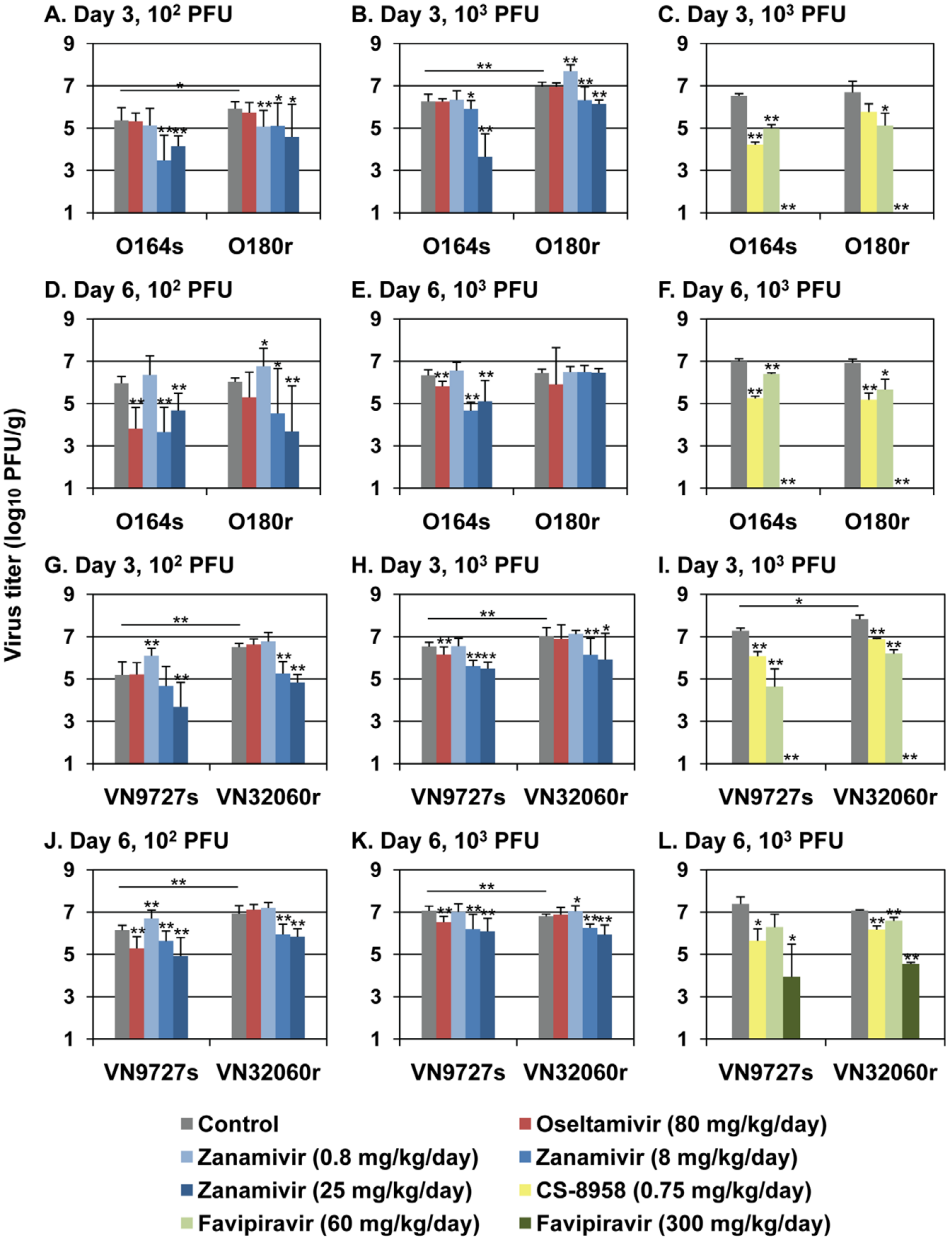
Antiviral sensitivity of viruses in mice. Mice were intranasally inoculated with 10^2^ (A, D, G, and J) or 10^3^ PFU of O164s, O180r (A–F), VN9727s, or VN32060r (G–L). At one hour post-infection (pi), mice were administered oseltamivir, zanamivir, CS-8958, favipiravir, or distilled water and PBS (control). Ten (A, B, D, E, G, H, J, and K) or three (C, F, I, and L) mice per group were euthanized on days 3 and 6 pi and virus titers in the lungs were determined by plaque assays in MDCK cells (detection limit: 1.0 log_10_ PFU/g). Error bars indicate the standard deviations of viral titers detected in the lungs of ten or three mice. Statistical significance was assessed by use of the Student's t-test: *, p<0.05, **, p<0.01.

To evaluate the effect of the NA H274Y substitution on the replication of the 2009 pandemic viruses *in vivo*, we infected BALB/c mice with either 10^2^ or 10^3^ plaque-forming units (PFU) of O164s, VN9727s, O180r, or VN32060r and determined virus titers in the lungs of mice on days 3 and 6 post-infection (pi) (Fig. 1, see “Control” samples). We found that oseltamivir-resistant viruses replicated significantly better than oseltamivir-sensitive viruses at both day 3 and day 6 pi with one exception: on day 6 pi, the virus titer in the lungs of mice infected with 10^3^ PFU of VN9727s was significantly higher than that of VN32060r-infected mice. These results indicate that the NA H274Y substitution does not negatively affect virus replication in the mouse lung in most settings.

Next, we tested virus sensitivity to anti-influenza drugs in mice (Fig. 1). One hour pi, mice received oseltamivir, zanamivir, CS-8958, or an experimental viral RNA polymerase inhibitor favipiravir (also known as T-705). When mice were treated with oseltamivir (80 mg/kg/day dose), the replication of the NA H274Y-possessing viruses O180r and VN32060r was not inhibited, whereas the virus titers of their oseltamivir-sensitive counterparts O164s and VN9727s were significantly reduced. These results support the results of the *in vitro* NA inhibition assays and growth kinetics in MDCK cells indicates that the NA H274Y substitution confers oseltamivir resistance to viruses in mice. Although the efficacy of zanamivir against an oseltamivir-resistant virus (O180r) was not prominent when mice were infected with 10^3^ PFU of viruses (Fig. 1), at a lower infectious dose (10^2^ PFU) of viruses, its efficacy was apparent. CS-8958 and favipiravir significantly reduced the replication of all viruses tested, indicating that these 2009 H1N1 pandemic isolates, including oseltamivir-resistant strains, are as susceptible to these experimental antiviral drugs as one of the initial isolates A/California/04/2009 (H1N1) [2].

### Pathogenicity and transmissibility of viruses in ferrets

To assess the transmissibility of oseltamivir-resistant 2009 H1N1 pandemic viruses, on day 1 pi we co-housed naïve ferrets in perforated cages next to ferrets inoculated with 10^6^ PFU of O164s, VN9727s, O180r, or VN32060r and collected nasal swabs from inoculated and contact ferrets on days 1, 3, 5, 7, 9, and 11 after infection and co-housing, respectively, for virological analysis (Table 2). For all viruses tested, all contact ferrets were infected with viruses via respiratory droplets from infected ferrets, although the detection of O180r in contact ferrets was delayed by up to two days in all three pairs. Sequencing of the NA gene of the oseltamivir-resistant viruses (i.e., O180r and VN32060r) revealed that all of the viruses isolated from the contact ferrets retained the oseltamivir resistance-conferring tyrosine at position 274 in NA. Collectively, there were no substantial differences in transmissibility between oseltamivir-sensitive and -resistant viruses in ferrets.

**Table 2:** Virus titers in the nasal swabs of inoculated and contact ferrets^a^.

	Virus titer (log10 PFU/ml) in animals infected with:																							
	O164s						O180r						VN9727s						VN32060r					
	Pair 1		Pair 2		Pair 3		Pair 4		Pair 5		Pair 6		Pair 7		Pair 8		Pair 9		Pair 10		Pair 11		Pair 12	
	*i*	*c*	*i*	*c*	*i*	*c*	*i*	*c*	*i*	*c*	*i*	*c*	*i*	*c*	*i*	*c*	*i*	*c*	*i*	*c*	*i*	*c*	*i*	*c*
Day 1 b	7.1^c^	-d	6.1	-	5.9	-	4.7	-	4.3	-	5.8	-	7.1	2.9	6.4	2.3	6.6	2.6	4.8	2.5	5.4	2.1	5.6	4.8
Day 3	5.1	1	4.5	3.7	4.2	2.0	3.8	-	3.0	-	2.0	-	5.2	7.4	4.6	5.3	4.8	5.8	4.2	5.8	4.4	4.9	6.1	5.2
Day 5	3.7	5.7	2.1	4.7	2.8	6.1	3.1	6.4	3.2	5.4	2.7	3	4.8	4.8	4.8	3.1	4.6	5.1	1.8	4.2	4.4	4.8	2.6	4.1
Day 7	-	-	-	5.5	-	4.1	-	5.1	-	4.7	-	5.3	-	3.6	-	-	1.3	-	-	2.3	4.4	1.8	-	1.7
Day 9	-	-	-	-	-	-	-	1.8	-	3	-	4.7	-	-	-	-	-	-	-	-	-	-	-	-
Day 11	-	-	-	-	-	-	-	-	-	-	-	-	-	-	-	-	-	-	-	-	-	-	-	-

a For pairs of ferrets, one animal was intranasally inoculated with 10^6^ PFU of virus (500 µl) (inoculated ferret, *i*) and one day later, a naïve ferret was placed in an adjacent cage (contact ferret, *c*). Nasal swabs were collected from all animals every other day for virus titration.

b Day 1 of co-housing of contact animals corresponds to Day 2 of infection.

c Virus titers in the nasal swabs were determined by plaque assays in MDCK cells.

d -, virus not detected (detection limit: 1.0 log_10_ PFU/g).

**Figure 2:**
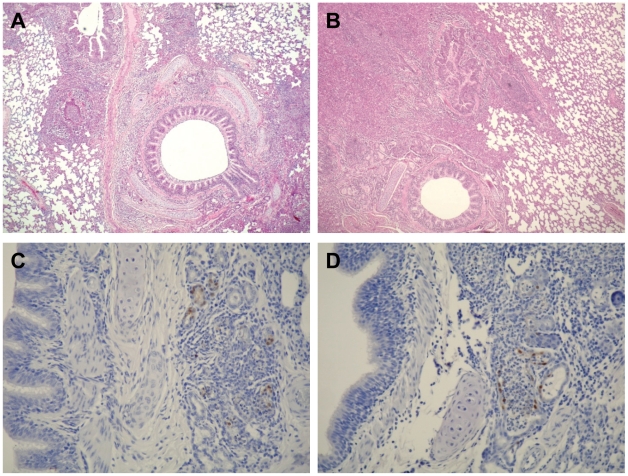
Pathological findings in the lungs of infected ferrets. Ferrets intranasally inoculated with 10^6^ PFU of O164s, O180r, VN9727s, or VN32060r were euthanized on day 6 pi. The nasal turbinates, lungs, and trachea were subjected to pathological examination. (A) Bronchopneumonia in a ferret infected with VN9727s (H&E staining). (B) Bronchopneumonia in a ferret infected with VN32060r (H&E staining). (C) Viral antigen (brown pigment) in the lungs of a ferret infected with O164s (immunohistological staining). (D) Viral antigen (brown pigment) in the lungs of a ferret infected with O180r (immunohistological staining).

To evaluate the pathogenicity of NA H274Y-possessing oseltamivir-resistant 2009 H1N1 pandemic viruses, we infected ferrets with 10^6^ PFU of O164s, VN9727s, O180r, or VN32060r and monitored them daily for changes in body temperature and weight, and for clinical signs. On day 6 pi, nasal turbinates, trachea, and lungs were removed for pathological analysis. All viruses tested caused respiratory symptoms such as sneezing, although no marked changes in body temperature or weight were observed (data not shown). We found no remarkable pathological differences in the extent and manifestation of disease between ferrets infected with oseltamivir-sensitive or -resistant viruses (Fig. 2). All ferrets examined had severe inflammatory changes in their respiratory tracts: rhinitis, tracheitis and bronchopneumonia with bronchadenitis (Fig. 2A and B). In addition, we found limited bronchadenitis lesions with viral antigens in four ferrets, two of which were infected with oseltamivir-sensitive viruses (either O164s or VN9727s) while the other two were infected with oseltamivir-resistant VN32060r (Fig. 2C and D). These results indicate the comparable pathogenicity of the oseltamivir-resistant viruses to their oseltamivir-sensitive counterparts in ferrets. These results also suggest that the oseltamivir-resistant 2009 H1N1 pandemic viruses have the potential to be readily transmitted among humans without loss of pathogenicity.

Here, we investigated antiviral sensitivity, pathogenicity, and transmissibility of 2009 H1N1 pandemic influenza viruses possessing the substitution NA H274Y, which is known to reduce the sensitivity of influenza viruses to oseltamivir in cultured cells, mice, and ferrets. Viruses with this NA substitution exhibited reduced sensitivity to oseltamivir with comparable replication, pathogenicity, and transmissibility compared to their oseltamivir-sensitive counterparts: these findings need to be validated in humans. The widespread administration of oseltamivir, and to a lesser extent zanamivir, will clearly contribute to the emergence of NA inhibitor-resistant viruses that retain optimal replication fitness and transmissibility in humans. Given our finding that the oseltamivir-resistant 2009 H1N1 pandemic viruses are as pathogenic and transmissible as their drug-sensitive counterparts, specific criteria for the use of NA inhibitors may need to be reconsidered.

Oseltamivir has been used extensively in patients with 2009 pandemic influenza, especially those who are immunocompromised. Approximately one-third of the oseltamivir-resistant pandemic strains have been isolated from immunocompromised patients (http://www.who.int/csr/disease/swineflu/frequently_asked_questions/antivirals/resistance/en/index.html), a possibility we predicted early in this pandemic [16]. Oseltamivir-resistant viruses readily emerge in immunocompromised patients partly because viruses can replicate to higher titers and for longer periods in immunocompromised hosts compared to those who are immunocompetent hosts [17]. Since the oseltamivir-resistant 2009 pandemic viruses are as pathogenic and transmissible as their drug-sensitive parents, immunocompromised influenza patients require special care - increased drug doses, combination therapy, and possibly isolation. In particular, combination therapy has the potential to reduce the emergence of drug-resistant mutants [18] and provide synergistic effects on the inhibition of virus replication [19]–[21] with no increase in adverse events [22]. Adopting such measures would help prevent the situation experienced with the oseltamivir-resistant seasonal H1N1 virus, which emerged during the 2007-2008 influenza season in Europe [23], spread, and superseded previously circulating oseltamivir-sensitive viruses [24], [25].

Finally, our *in vitro* and *in vivo* experiments suggest that the experimental drugs CS-8958 and favipiravir may be promising candidates to combat 2009 H1N1 pandemic viruses.

## Materials and Methods

### Viruses and cells

We used two pairs of H1N1 pandemic virus clinical isolates; A/Osaka/164/2009 (O164s), A/Osaka/180/2009 (O180r), A/Vietnam/HCM9727/2009 (VN9727s), and A/Vietnam/HN32060/2009 (VN32060r). All of the viruses were isolated in Madin-Darby canine kidney (MDCK) cells, which were maintained in Eagle’s minimal essential medium (MEM) supplemented with 5% newborn calf serum (Sigma, St. Louis, MO) and cultured at 35°C in 5% CO_2_. Tyrosine at position 275 in the NA of O180r and VN32060r was confirmed by molecular cloning and sequencing of 18 individual clones each, while the rest of the viral genome sequences were determined by direct sequencing of amplified DNA.

### Antiviral compounds

Oseltamivir (oseltamivir phosphate) and oseltamivir carboxylate (the active metabolite of oseltamivir; GS4104) were prepared from Tamiflu (Roche Laboratories Inc., Basel, Switzerland). Zanamivir, CS-8958 (an experimental NA inhibitor), and R-125489 (the active metabolite of CS-8958) were synthesized at Daiichi Sankyo Co. Ltd. according to published procedures [26]. Favipiravir (an experimental broad-spectrum viral RNA polymerase inhibitor, also known as T-705), was synthesized at Toyama Chemical Co., Ltd.

### NA inhibition assay

NA activity of viruses in the presence of NA inhibitor was measured by an NA inhibition assay as described previously [8], [27], [28]. Briefly, diluted viruses were mixed with various concentrations of NA inhibitor in 2-[N-morpholino]ethanesulfonic acid containing CaCl_2_ and incubated for 30 min at 37°C. A fluorescent substrate methylumbelliferyl-N-acetylneuraminic acid (Sigma) was added to this mixture, which was then incubated for one hour at 37°C. After adding NaOH in 80% ethanol to the mixture to stop the reaction, the fluorescence of the solution was measured at an excitation wavelength of 360 nm and an emission wavelength of 465 nm and the 50% inhibitory concentration (IC_50_) was calculated.

### *In vitro* growth kinetics

MDCK cells were infected with viruses at a multiplicity of infection of 0.0001. One hour later, oseltamivir carboxylate, zanamivir, R-125489 (10 µM each), or nothing (control) was added to the cells. At 8, 16, 24, 32, 40, and 48 h post-infection (pi), the supernatants of the infected cells were harvested and subjected to plaque assays in MDCK cells to determine virus titers.

### Experimental infection of mice

Six-week-old female BALB/c mice (Japan SLC Inc., Shizuoka, Japan) were anesthetized with isoflurane and intranasally infected with 10^2^ or 10^3^ plaque-forming units (PFU) (50 µl) of viruses. One hour pi, six mice per group were administered the following antiviral compounds: (1) oseltamivir phosphate: 80 mg per kg per 400 µl (divided into two oral administrations per day) for 5 days; (2) zanamivir: 0.8, 8, or 25 mg per kg per 50 µl in one daily intranasal administration for 5 days; (3) CS-8958: 0.75 mg per kg per 50 µl in one intranasal administration; (4) Favipiravir: 60 or 300 mg per kg per 400 µl (divided into two oral administrations per day) for 5 days; (5) or distilled water orally (200 µl) and PBS intranasally (50 µl). Three mice per group were euthanized on days 3 and 6 pi and virus titers in the lungs were determined by plaque assays in MDCK cells.

### Experimental infection of ferrets

Three-to-four-month-old male ferrets (Marshall Farms, Wolcott, NY) and six-to-eight-month-old female ferrets (Triple F Farms, Sayre, PA) were used; animals from Triple F Farms were used for the transmission study of O164s and O180r, while those from Marshall Farms were used for the rest of studies. All of the animals, which were serologically negative for currently circulating human influenza viruses (including pandemic H1N1 viruses) by haemagglutination inhibition (HI) assay, were anesthetized with ketamine and xylazine (5 mg and 0.5 mg per kg of body weight, respectively) and intranasally infected with 10^6^ PFU (500 µl) of viruses. On day 6 pi, three ferrets per group were euthanized and their nasal turbinates, trachea, and lungs subjected to virological and pathological analyses. For the transmission study, on day 1 pi, three naïve ferrets per group were co-housed in a perforated cage adjacent to an inoculated ferret; ferrets were separated by a perforated divider and did not have direct contact. All ferrets were monitored daily for changes in body temperature and weight, and clinical signs. Nasal swabs of inoculated and contact ferrets were collected on days 1, 3, 5, 7, 9, and 11 after infection and co-housing, respectively, and virus titers were determined by plaque assays in MDCK cells.

### Pathological examination

Nasal turbinates, lungs, and trachea of euthanized ferrets were preserved in 10% phosphate-buffered formalin. Tissues were then processed for paraffin embedding and cut into 5 µm-thick sections. One section from each tissue sample was subjected to standard hematoxylin-and-eosin (H&E) staining, while another was processed for immunohistological staining with an anti-influenza virus rabbit antibody (R309; prepared in our laboratory) that reacts comparably with all viruses tested. Specific antigen-antibody reactions were visualized by 3,3'-diaminobenzidine tetrahydrochloride staining using a Dako EnVision system (Dako Co. Ltd., Tokyo, Japan).

### Ethics

Our research protocol for the use of mice and ferrets followed the University of Tokyo's Regulations for Animal Care and Use, which was approved by the Animal Experiment Committee of the Institute of Medical Science, the University of Tokyo (approval number: 19–28). The committee acknowledged and accepted both the legal and ethical responsibility for the animals, as specified in the Fundamental Guidelines for Proper Conduct of Animal Experiment and Related Activities in Academic Research Institutions under the jurisdiction of the Ministry of Education, Culture, Sports, Science and Technology, 2006.

## Supporting Information

Figure S1 Growth kinetics of viruses in MDCK cells. Cells were infected with O164s (A), O180r (B), VN9727s (C), or VN32060r (D) at a multiplicity of infection of 0.0001. One hour later, oseltamivir carboxylate, zanamivir, R-125489 (10 µM each), or nothing (control) was added to the cells. The virus titers in the supernatants at the indicated times post-infection were assessed by plaque assays in MDCK cells. Error bars indicate the standard deviations of the viral titers from triplicate experiments.
(0.27 MB TIF)

Table S1 Amino acid differences among the viruses tested.
(0.16 MB DOC)
