# Supplementary figures and images for: Characterization of Oseltamivir-Resistant 2009 H1N1 Pandemic Influenza A Viruses
Source: PLoS Pathog. 2010 Aug 26;6(8):e1001079. doi: 10.1371/journal.ppat.1001079 (PMC2928817; doi:10.1371/journal.ppat.1001079)

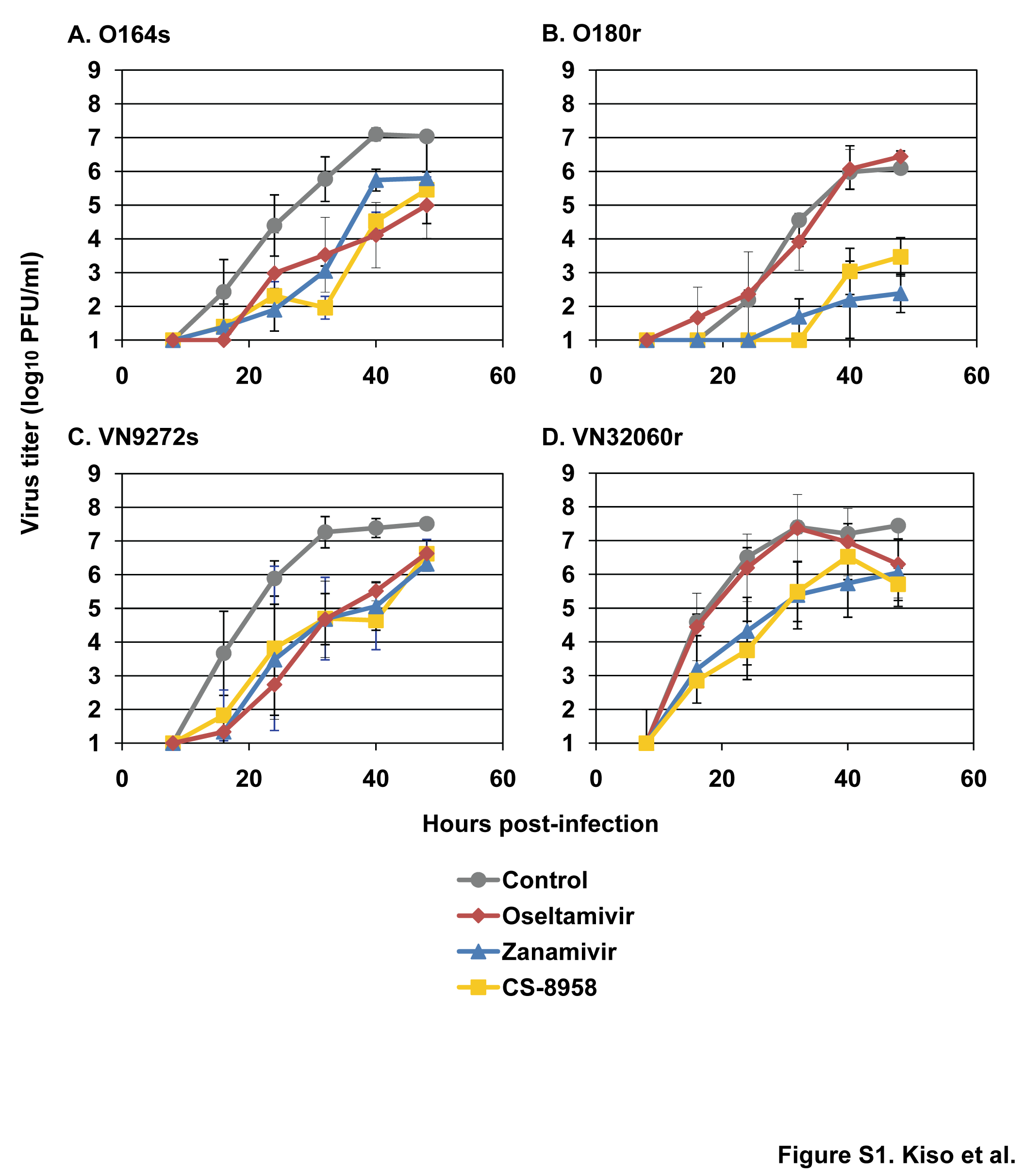

Supplement: Figure S1 — Growth kinetics of viruses in MDCK cells. Cells were infected with O164s (A), O180r (B), VN9727s (C), or VN32060r (D) at a multiplicity of infection of 0.0001. One hour later, oseltamivir carboxylate, zanamivir, R-125489 (10 µM each), or nothing (control) was added to the cells. The virus titers in the supernatants at the indicated times post-infection were assessed by plaque assays in MDCK cells. Error bars indicate the standard deviations of the viral titers from triplicate experiments. (0.27 MB TIF) [file ppat.1001079.s001.tif]
