# Supplementary material for: Characterization of Oseltamivir-Resistant 2009 H1N1 Pandemic Influenza A Viruses
Source: PLoS Pathog. 2010 Aug 26;6(8):e1001079. doi: 10.1371/journal.ppat.1001079 (PMC2928817; doi:10.1371/journal.ppat.1001079)
Supplement: Table S1 — Amino acid differences among the viruses tested. (0.16 MB DOC) [file ppat.1001079.s002.doc]

**Supplementary Table S1. Amino acid differences among the viruses tested.**

| Protein | Position | Amino acid for indicated strain | | | | |
| --- | --- | --- | --- | --- | --- | --- |
|  |  | O164s | O180r |  | VN9727s | VN32060r |
| PB2 | 649 | I | I |  | V | V |
|  | 700 | E | K |  | E | E |
| PB1 | 667 | T | T |  | I | I |
| HA1 | 152 | V | I |  | V | V |
|  | 203 | S | S |  | T | T |
| NA | 248 | N | N |  | D | D |
|  | 275 | H | Y |  | H | Y |
| M1 | 33 | A | T |  | A | A |
| NS1 | 115 | L | L |  | L | F |
|  | 123 | I | I |  | V | V |
| NS2 | 63 | K | K |  | E | E |
